# [Retracted] 2,3,5,4‑tetrahydroxy diphenylethylene‑2‑O‑glucoside inhibits the adhesion and invasion of A549 human lung cancer cells

**DOI:** 10.3892/mmr.2024.13260

**Published:** 2024-06-10

**Authors:** Ming Xu, Cong Wang, Minglin Zhu, Xianguo Wang, Li Zhang, Jinping Zhao

Mol Med Rep 16: 8900–8906, 2017; DOI: 10.3892/mmr.2017.7680

Following the publication of this paper, it was drawn to the Editor's attention by a concerned reader that the data featured in Figs. 1, 4 and 5 (including western blotting data) were strikingly similar to data that had appeared in a different form in a different article by different authors at different research institutes.

Owing to the fact that the contentious data in the above article had already been accepted for publication in another article prior to its submission to *Molecular Medicine Reports*, the Editor has decided that this paper should be retracted from the Journal. The authors were asked for an explanation to account for these concerns, but the Editorial Office did not receive a reply. The Editor apologizes to the readership for any inconvenience caused.

